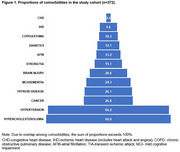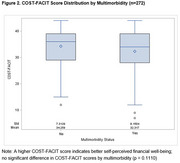# Exploring the Intersection of Multimorbidity and Financial Toxicity Among Older Adults: Findings from the UK‐ADRC Cohort

**DOI:** 10.1002/alz70860_097255

**Published:** 2025-12-23

**Authors:** Rada Choate, Justin M Barber, Gregory A Jicha, Erin L. Abner

**Affiliations:** ^1^ College of Public Health, University of Kentucky, Lexington, KY, USA; ^2^ Sanders‐Brown Center on Aging, University of Kentucky, Lexington, KY, USA; ^3^ University of Kentucky College of Medicine, Lexington, KY, USA; ^4^ University of Kentucky Sanders‐Brown Center on Aging, Lexington, KY, USA; ^5^ Department of Epidemiology and Environmental Health, University of Kentucky, Lexington, KY, USA

## Abstract

**Background:**

Older adults frequently experience multimorbidity, defined as the coexistence of multiple chronic diseases. In ADRD, multimorbidity is associated with poor physical and mental health outcomes, accelerated cognitive decline, increased mortality, and substantial economic burden, including direct healthcare costs and indirect costs from lost productivity and informal caregiving. While the concept of “financial toxicity” is well‐studied in cancer care, its impact on individuals living with ADRD and multimorbidity remains underexplored.

**Methods:**

This study analyzed data from adults aged ≥50 years enrolled in the University of Kentucky Alzheimer's Disease Research Center (UK‐ADRC) cohort, a convenience sample of community‐dwelling older adults across the spectrum of cognition followed longitudinally until death. Multimorbidity was assessed based on the presence of 12 measured chronic conditions (Figure 1). Financial toxicity was measured using the **Co**mprehensive **S**core for Financial **T**oxicity (COST‐FACIT) v.2 instrument (11‐items, score range 0‐44), with higher scores indicating better financial well‐being. The instrument was adapted for individuals with normal cognition, mild cognitive impairment, and dementia. Descriptive statistics and unadjusted and adjusted linear regression models were used to examine the association between multimorbidity and self‐perceived financial distress.

**Results:**

Among the 272 participants (median age 78 [IQR:73‐83], 63% female, 92% white, median education of 17 years [IQR:16‐18], 76% had normal cognition, 16% had MCI, and 8% had dementia. Multimorbidity was present in 80% of the cohort, with a median of 3 conditions per individual (IQR:2‐4). The median COST‐FACIT score was 35 (IQR:28‐39), indicating low overall financial burden in this cohort, with no significant difference between individuals with and without multimorbidity (Figure 2). However, multivariable analyses showed that higher COST‐FACIT scores (better financial well‐being) were significantly associated with fewer comorbidities, older age, and higher educational attainment.

**Conclusions:**

This study found that multimorbidity was prevalent among older adults in this cohort, and better financial well‐being was linked to fewer comorbidities, older age, and higher educational attainment. Future research and larger studies with more participants with MCI and dementia are needed to investigate the nuanced interplay between patterns of multimorbidity and financial burden, coping strategies, and health‐related quality of life in older adults with ADRD and their families.